# RETRACTION: lncRNA PANTR1 Upregulates BCL2A1 Expression to Promote Tumorigenesis and Warburg Effect of Hepatocellular Carcinoma through Restraining miR-587

**DOI:** 10.1155/jimr/9874823

**Published:** 2025-05-04

**Authors:** Journal of Immunology Research

RETRACTION: X. Ma, Z. Mao, J. Zhu, H. Liu, F. Chen, lncRNA PANTR1 Upregulates BCL2A1 Expression to Promote Tumorigenesis and Warburg Effect of Hepatocellular Carcinoma through Restraining miR-587, *Journal of Immunology Research*, 2021. https://doi.org/10.1155/2021/1736819

The above article, published online on 13 August 2021 in Wiley Online Library (wileyonlinelibrary.com), has been retracted by agreement between the Editorial Board; and John Wiley & Sons Ltd. The retraction has been agreed due to concerns initially raised by the authors about the reliability of the results presented. In a subsequent investigation by the journal, inconsistencies were identified in Figure 5G, raising further concerns about the reliability of the data and necessitating its retraction.

